# Novel insertion/deletion polymorphisms and genetic features of the shadow of prion protein gene (*SPRN*) in dogs, a prion-resistant animal

**DOI:** 10.3389/fvets.2022.942289

**Published:** 2022-08-02

**Authors:** Yong-Chan Kim, Hyeon-Ho Kim, An-Dang Kim, Byung-Hoon Jeong

**Affiliations:** ^1^Korea Zoonosis Research Institute, Jeonbuk National University, Iksan, South Korea; ^2^Department of Bioactive Material Sciences and Institute for Molecular Biology and Genetics, Jeonbuk National University, Jeonju, South Korea; ^3^Cool-Pet Animal Hospital, Anyang, Gyeonggi, South Korea

**Keywords:** dogs, prion, *PRNP*, *SPRN*, polymorphism, canine, Sho

## Abstract

Prion diseases are fatal infectious neurodegenerative disorders that are induced by misfolded prion protein (PrP^Sc^). Previous studies have reported that the shadow of prion protein (Sho) encoded by the shadow of prion protein gene (*SPRN*) plays a critical role in stimulating the conversion process of normal PrP (PrP^C^) into PrP^Sc^, and genetic polymorphisms of the *SPRN* gene are significantly related to susceptibility to prion diseases. Recent studies have reported that dogs show prion resistance, and there have been several attempts to identify resistance factors to prion diseases in dogs. However, there has been no study of the canine *SPRN* gene thus far. We investigated genetic polymorphisms of the canine *SPRN* gene in 201 dogs using amplicon sequencing and compared the number of *SPRN* polymorphisms among prion-related species. In addition, we performed multiple sequence alignments of the amino acid sequences of Sho among prion-related species by ClustalW and analyzed the 3D structure of Sho using AlphaFold. Furthermore, we assessed the protein–protein interaction of canine PrP with canine Sho carrying wild-type and mutant alleles using HawkDock. We found four novel insertion/deletion polymorphisms of the *SPRN* gene in 201 dogs and identified a significant difference in the number of *SPRN* polymorphisms between prion-susceptible and prion-resistant animals. In addition, Sho has two α-helixes linked with the coil. Furthermore, we found different binding complexes and binding free energies between canine Sho and PrP according to *SPRN* polymorphisms. To the best of our knowledge, this is the first report of canine *SPRN* polymorphisms.

## Introduction

Prion diseases are fatal and infectious disorders that are reported in a wide range of hosts, including humans, cattle, sheep, goats, deer, and elk. Prion diseases occur by misfolded prion protein (PrP^Sc^) originating from normal prion protein (PrP^C^) (1–3). To date, although the conversion process of PrP^C^ to PrP^Sc^ has been elusive, several cofactors that play a critical role in the exacerbation of prion diseases have been identified (4–9). Among these cofactors, the shadow of prion protein (Sho) encoded by the shadow of prion protein gene (*SPRN*) stimulates the conversion process of PrP^C^ to PrP^Sc^ *via* interaction with PrP (7). Thus, previous studies have reported that genetic polymorphisms of the *SPRN* gene that affect the expression level, function, and structure of Sho are involved in susceptibility to several types of prion diseases. In humans, a null allele of the *SPRN* gene was observed in variant Creutzfeldt–Jakob disease (vCJD) patients (10). In addition, the insertion polymorphism in the 3' untranslated region (UTR) of the caprine *SPRN* gene is related to susceptibility to scrapie in goats (11). Furthermore, rare sequence variation in the open reading frame (ORF) of the bovine *SPRN* gene was found in L-type atypical bovine spongiform encephalopathy (BSE)-affected cattle (12). These studies indicate a clear association of Sho with pathomechanisms in various prion diseases. In recent studies, prion diseases have not been reported in dogs during the 1986 BSE outbreak. Thus, there have been several attempts to identify resistance to prion diseases in dogs (13–17). To date, Sho, a critical factor in PrP^Sc^ conversion, has not been analyzed. Furthermore, as Sho has an unstructured 3D structure, comparative analysis of the 3D structure of Sho in prion-related animals has not been investigated thus far.

In the present study, we investigated genetic polymorphisms of the canine *SPRN* gene in 201 dogs using amplicon sequencing and compared the number of *SPRN* polymorphisms among prion-related species. In addition, we compared amino acid sequences of several prion-related species to find unique genetic characteristics of canine Sho by ClustalW and predicted the 3D structure of Sho of several prion-related species using AlphaFold (18, 19). Furthermore, we analyzed the protein–protein interaction of canine PrP with canine Sho carrying wild-type and mutant alleles using HawkDock (20).

## Materials and methods

### Ethical statements

All experimental protocols were approved by the Institute of Animal Care and Use Committee of Jeonbuk National University (JBNU 2018-062).

### Samples

The whole-blood samples were byproducts of completing blood count to evaluate overall health and testing several diseases, including anemia, infection, and leukemia in dogs and were collected from 201 dogs by a qualified veterinarian. The samples were provided by Anyang Cool-Pet Animal Hospital in South Korea. The dogs consisted of eight dog breeds. Detailed information on the dog breeds is provided in Supplementary Table 1. The samples were obtained depending upon hospital visitation based on the most common dog breed in the South Korea. The dog breed was determined by a pedigree document.

### Genomic DNA extraction

Genomic DNA was extracted from whole-blood samples using the Labopass Blood Genomic DNA Isolation Kit Mini (Cosmogenetech, Seoul, Korea) following the method provided by the manufacturer. The quality and concentration of genomic DNA samples (15–20 ng/μl, A260/280 = 1.8–2.0) were assessed by Nanodrop (Thermo Scientific, Waltham, USA).

### Amplification of the canine *SPRN* gene

The canine *SPRN* gene (Gene ID: 100855950) was amplified from genomic DNA by polymerase chain reaction (PCR) using gene-specific primers (F: 5′-AAGCTGAGCCCTTTCCCCTT-3′, R: 5′-CAGGGCACCTGCCTCTCT-3′). PCR was performed using a C1000 Touch Thermal Cycler (Bio–Rad, Hercules, California, USA) and H-star *Taq* DNA polymerase (BIOFACT, Daejeon, Korea) following the manufacturer's recommendations. The experimental PCR conditions were as follows: 98°C for 15 min for denaturation; 34 cycles of 98°C for 20 s, 60°C for 40 s, and 72°C for 1 min for annealing and extension; and one cycle of 72°C for 5 min for the final extension. The PCR products were analyzed by an ABI 3,730 sequencer (ABI, Forster City, CA, USA), and the electropherograms of the sequencing peaks were read by Finch TV software (Geospiza Inc., Seattle, WA, USA).

### Statistical analyses

Linkage disequilibrium (LD) and haplotype analyses were performed using Haploview version 4.2 (Broad Institute, Cambridge, MA, USA).

### Multiple sequence alignments

The amino acid sequences of Sho were collected from GenBank at the National Center for Biotechnology Information (NCBI). Detailed information on Sho is described in Supplementary Table 2. The sequences were compared by ClustalW based on progressive alignment methods.

### PROVEAN

PROVEAN (http://provean.jcvi.org/seq_submit.php) was used to evaluate the effect of amino acid substitution on the structure/function of proteins. PROVEAN assigns scores by calculating BLAST hits of homologs extracted from the NCBI. PROVEAN scores were classified as follows: above −2.5 indicates “neutral,” and below −2.5 indicates “deleterious.”

### AlphaFold

The 3D structure of Sho was predicted by AlphaFold based on machine learning (https://colab.research.google.com/github/deepmind/alphafold/blob/main/notebooks/AlphaFold.ipynb). The confidence of modeling was assessed by the predicted local distance difference test (pLDDT) score on a scale from 0 to 100.

### HawkDock

Protein–protein interactions and complex structures were predicted by HawkDock based on the docking algorithm “ATTRACT”. MM/GBSA calculated the structure evaluation of protein–protein interactions and binding free energy. The 3D structure of canine PrP (PDB ID: 1XYK) was deposited as the receptor. 3D structures of canine Sho with wild-type and mutant alleles were generated by AlphaFold and deposited as the ligands.

### TM-Align

The structural similarity of two query proteins was evaluated by TM-align based on heuristic dynamic programming iteration. TM-align calculated TM-score and average root mean square deviation (RMSD). TM-align scores below 0.2 indicate “randomly chosen unrelated protein.” TM-align scores above 0.5 indicate “in about the same fold.”

## Results

### Identification of four novel insertion/deletion polymorphisms of the canine *PRNP* gene in 201 dogs

**Figure 1:**
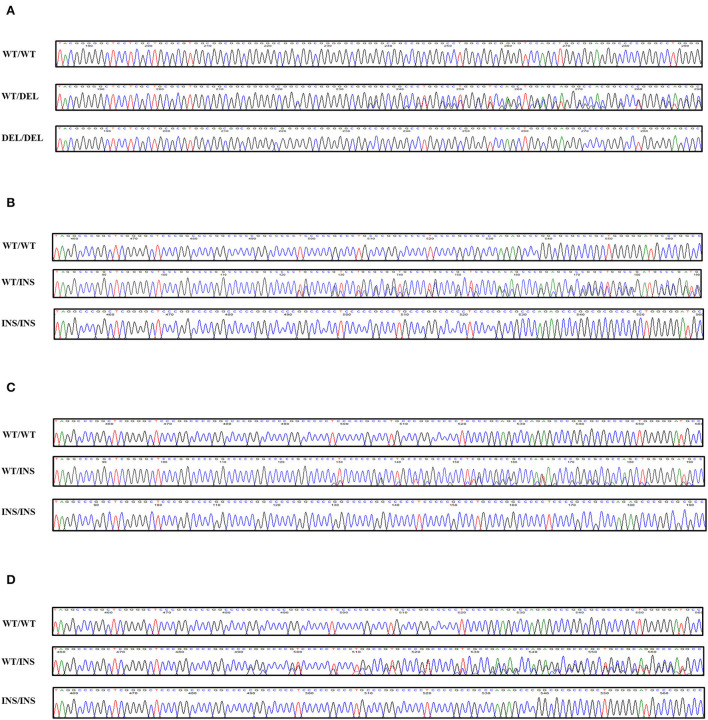
Novel genetic polymorphisms of the canine shadow of prion protein gene (*SPRN*) in the present study. **(A)** c.209_214DelCGGCGG (70_71DelAA). **(B)** c.444+30_444+31InsGGCCCCC. **(C)** c.444+30_444+31InsGGCCCCCGGCCCC. **(D)** c.444+31_444+32InsGGCCCC. Four colors show individual bases of the DNA sequence (blue: cytosine, red: thymine, black: guanine, green: adenine). WT, wild-type allele of the canine *SPRN* gene; DEL, deletion allele of the canine *SPRN* gene; INS, insertion allele of the canine *SPRN* gene.

To investigate canine *SPRN* gene polymorphisms, we performed PCR to amplify the ORF region of the canine *SPRN* gene. We identified four novel insertion/deletion polymorphisms, including c.209_214DelCGGCGG (70_71DelAA) in the ORF region and c.444+30_444+31InsGGCCCCC, c.444+30_444+31InsGGCCCCCGGCCCC, and c.444+31_444+32InsGGCCCC in the intron (Figure 1). The genotype and allele distributions of the insertion/deletion polymorphisms of the canine *SPRN* gene are described in Table 1. We also investigated the LD values among the three canine *SPRN* polymorphisms with *r*^2^ values (Table 2). Notably, strong LD (*r*^2^ >0.333) was found between c.444+30_444+31InsGGCCCCC and c.444+31_444+32InsGGCCCC. In addition, we performed a haplotype analysis of four polymorphisms of the canine *SPRN* gene (Table 3). The WT-WT-WT-Ins haplotype was most frequently observed (31.2%), followed by Del-Ins-WT-WT (22.3%) and WT-WT-WT-WT (22.2%) in the canine *SPRN* gene (Table 3).

**Table 1:** Genotype and allele frequencies of shadow of prion protein gene (*SPRN*) polymorphisms in 201 dogs.

**Polymorphisms**	**Location**	**Total, *n***	**Genotype frequencies**, ***n***			**Allele frequencies**, ***n***	
			**MM**	**Mm**	**mm**	**M**	**m**
c.209_214DelCGGCGG (70_71DelAA)	ORF	201	132	37	32	301	101
c.444+30_444+31InsGGCCCCC	3′UTR	201	107	15	79	229	173
c.444+30_444+31InsGGCCCCCGGCCCC	3′UTR	201	199	1	1	399	3
c.444+31_444+32InsGGCCCC	3′UTR	201	130	9	62	269	133

MM, major homozygote; Mm, heterozygote; mm, minor homozygote; M, major allele; m, minor allele; ORF, open reading frame; 3'UTR, 3' untranslated region.

**Table 2:** Linkage disequilibrium (LD) among genetic polymorphisms of the *SPRN* gene in dogs.

*r* ^2^	c.209_214DelCGGCGG	c.444+ 30_444+ 31InsGGCCCCC	c.444+ 30_444+ 31InsGGCCCCCGGCCCC	c.444+ 31_444+ 32InsGGCCCC
c.209_214DelCGGCGG	-		-	-
c.444+30_444+31InsGGCCCCC	0.287		-	-
c.444+30_444+31InsGGCCCCCGGCCCC	0.003	0.006	-	-
c.444+31_444+32InsGGCCCC	0.128	0.374	0.015	-

Bold text indicates strong LD (r^2^ > 0.333).

**Table 3:** Haplotype frequencies of 4 *SPRN* polymorphisms in dogs.

**Haplotypes**	**c.209_214DelCGGCGG**	**c.444+ 30_444+ 31InsGGCCCCC**	**c.444+30_444+ 31InsGGCCCCCGGCCCC**	**c.444+ 31_444+ 32InsGGCCCC**	**Dog (*n* = 402)**
Ht1	WT	WT	WT	Ins	125 (0.312)
Ht2	Del	Ins	WT	WT	90 (0.223)
Ht3	WT	WT	WT	WT	89 (0.222)
Ht4	WT	Ins	WT	WT	83 (0.207)
Ht5	Del	WT	WT	WT	9 (0.017)
Ht6	Del	WT	WT	Ins	4 (0.012)
Others	-	-	-	-	2 (0.007)

### Comparison of genetic polymorphisms of the *SPRN* gene among several species

**Figure 2:**
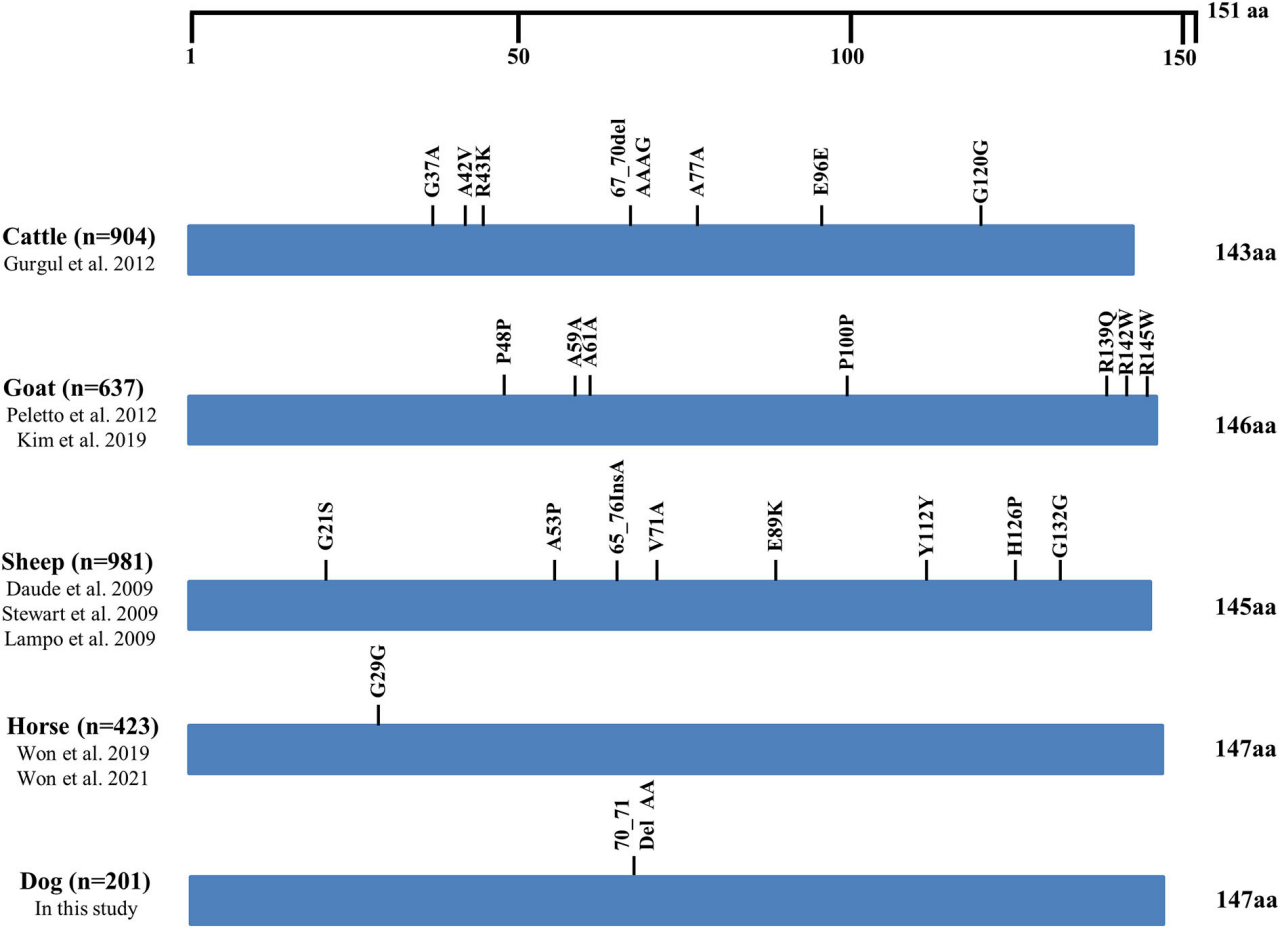
Distribution of genetic polymorphisms in the open reading frame (ORF) of the *SPRN* gene in several species. The figure indicates reported genetic polymorphisms of the *SPRN* gene in humans, cattle, goats, sheep, horses, and dogs. The edged horizontal bar indicates the length of the amino acids in the *SPRN* gene.

We compared the distributions of the genetic polymorphisms found in the ORF of the *SPRN* gene in prion disease-susceptible (human, cattle, goat, and sheep) and prion disease-resistant (horse and dogs) animals. Notably, prion disease-susceptible animals, including 904 cattle, 637 goats and 981 sheep showed over seven genetic polymorphisms of the *SPRN* gene (cattle: 7; goats: 7; sheep: 8). However, only one genetic polymorphism of the *SPRN* gene was observed in prion disease-resistant animals, including 423 horses and 201 dogs (Figure 2).

### Comparison of Sho of dogs with that of several species

**Figure 3:**
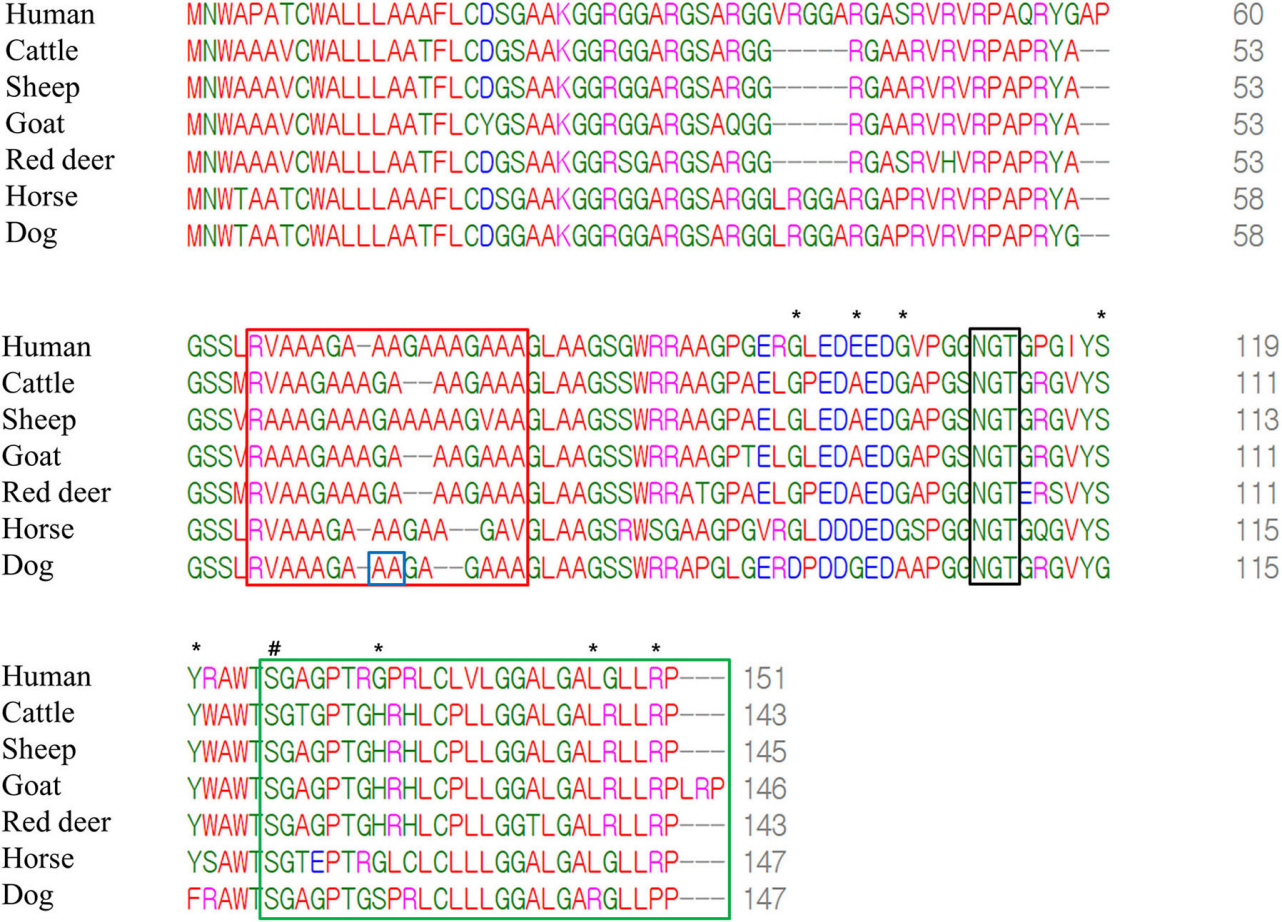
Multiple sequence alignments of amino acid sequences of the shadow of prion protein (Sho) in humans, cattle, sheep, goats, red deer, horses and dogs. The colors show the chemical properties of amino acids; blue: acidic; red: small and hydrophobic; magenta: basic; green: hydroxyl, sulfhydryl, amine and glycine. Asterisks indicate dog-specific amino acids. Sharp indicates the omega site of the glycosylphosphatidylinositol (GPI) anchor. The red box indicates the interaction region of Sho with prion protein (PrP). The black box indicates the NXT glycosylation motif of Sho. The green box indicates the signal sequence of the GPI anchor of Sho. The blue box indicates genetic polymorphisms identified in the present study.

We compared amino acid sequences of Sho among seven species, including humans, cattle, sheep, goats, red deer, horses, and dogs, using multiple sequence alignments (Figure 3). Notably, we found a high conservation of the PrP interaction domain of Sho (red box), the NXT glycosylation motif (black box), and the omega site (serine) and signal sequence (green box) of the C-terminal domain among all species. The canine Sho showed a total of eight canine-specific amino acids, including D95, G99, A102, G115, F116, S128, R142, and P146. In addition, equine Sho showed a total of 11 equine-specific amino acids, including R84, S86, G87, V93, D99, S103, Q111, S117, E124, L129, and C130. However, these codons were not in the same location as polymorphisms. Among several species, human Sho has the highest identity with canine Sho (80.8%) followed by equine Sho (80.3%), bovine Sho (77.2%), and ovine Sho (75.3%) (Supplementary Table 2).

### *In silico* evaluation of the effect according to canine Sho substitutions using PROVEAN

To evaluate the effect of the substitutions on the function of canine Sho, PROVEAN was used (Table 4). Notably, 70_71DelAA was predicted to be “deleterious” with a score of “−5.125.” However, all substitutions of canine-specific amino acids with interspecies conserved amino acids were predicted to be “neutral.”

**Table 4:** *In silico* evaluation of the effect according to the substitutions of the canine shadow of prion protein (Sho) using PROVEAN.

**Substitutions**	**PROVEAN**	
	**Score**	**Prediction**
70_71DelAA	−5.125	Deleterious
D95G	3.027	Neutral
G99E	1.620	Neutral
G99A	−0.066	Neutral
G99D	1.927	Neutral
A102G	1.691	Neutral
G115S	3.227	Neutral
F116Y	2.923	Neutral
S128G	0.661	Neutral
S128H	−0.550	Neutral
R142L	3.050	Neutral
P146R	3.168	Neutral

### Prediction of the 3D structure of Sho among various species using AlphaFold

**Figure 4:**
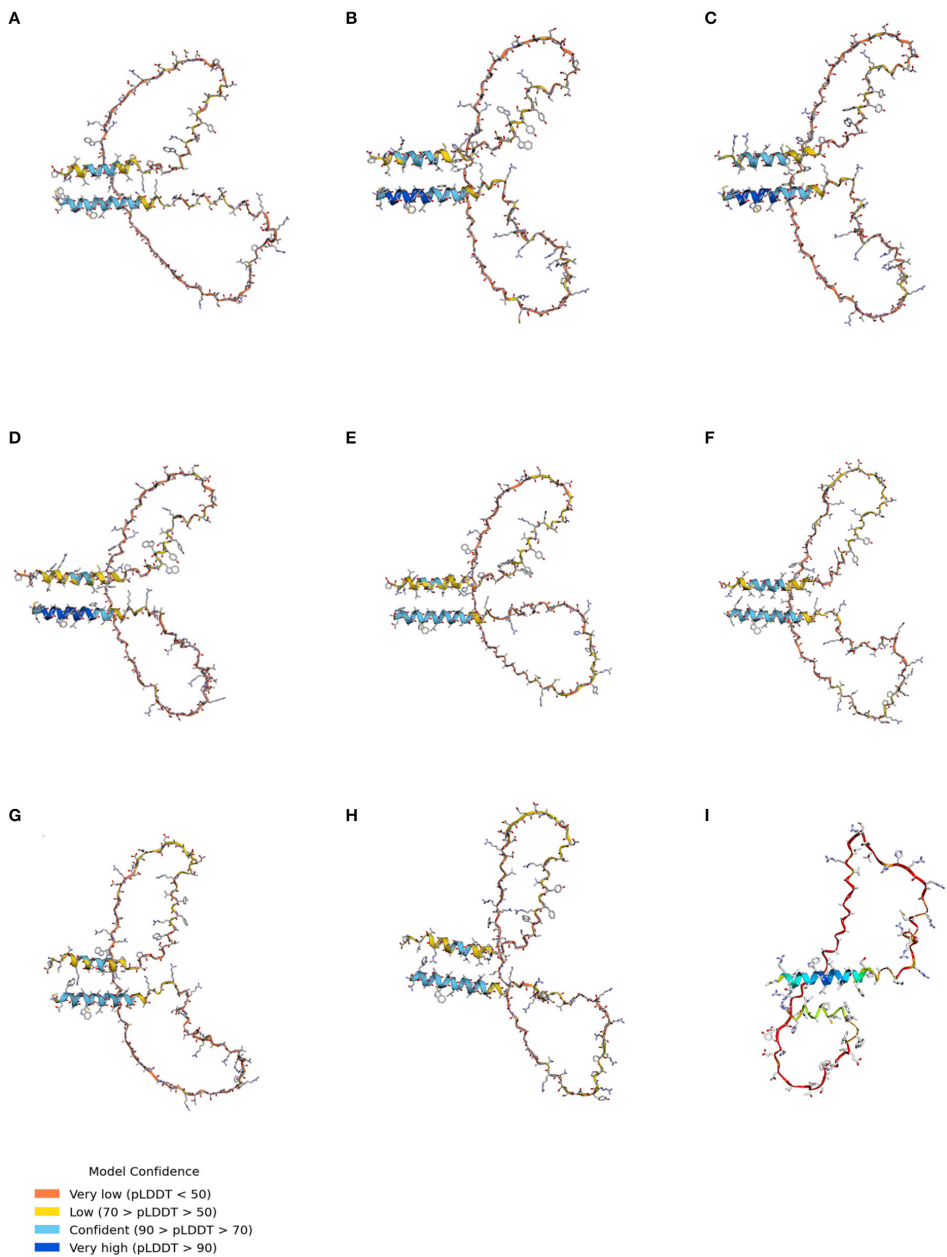
The tertiary structure of Sho. **(A)** The tertiary structure of the human Sho. **(B)** The tertiary structure of bovine Sho. **(C)** The tertiary structure of ovine Sho. **(D)** The tertiary structure of caprine Sho. **(E)** The tertiary structure of red deer Sho. **(F)** The tertiary structure of equine Sho. **(G)** The tertiary structure of canine Sho with the wild-type allele. **(H)** The tertiary structure of canine Sho with the 70_71DelAA allele. **(I)** The tertiary structure of chicken Sho. pLDDT, predicted local distance difference test.

AlphaFold was used to analyze the 3D structure of Sho among seven species, including humans, cattle, sheep, goats, red deer, horses, dogs, and chickens (Figure 4). Notably, all Sho was predicted to have two α-helixes linked with the coil (Figures 4A–I). In addition, we predicted the 3D structure of canine Sho carrying the 70_71DelAA allele. We did not find a significant difference between the canine Sho carrying the wild-type and the 70_71DelAA alleles (Figures 4G,H). We evaluated the structural similarity of wild-type Sho of dogs with Sho of several species using TM-align (Supplementary Table 3). All Sho proteins have a higher TM-align score above 0.2 and lower RMSD below 6Å. TM-align indicates that the wild-type Sho of dogs has a similar structure to Sho of humans, cattle, sheep, goats, red deer, and horses.

### Protein–protein interaction of canine Sho with canine PrP using HawkDock

**Figure 5:**
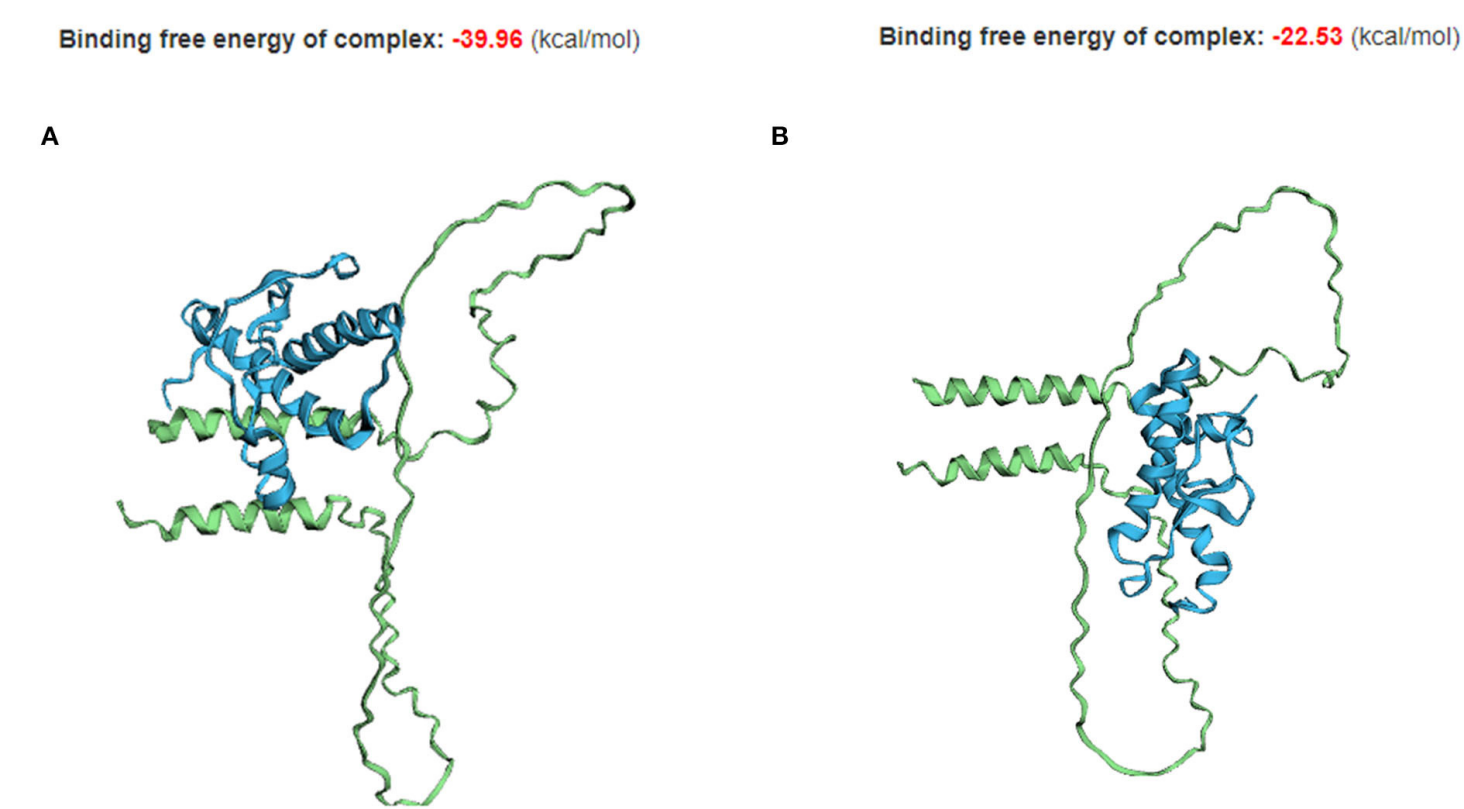
The tertiary structure and protein–protein interaction of canine PrP with canine Sho carrying the wild-type and 70_71DelAA allele. **(A)** Canine PrP interaction with canine Sho carrying the wild-type allele. **(B)** Canine PrP interaction with canine Sho carrying the 70_71DelAA allele. The blue structure indicates canine PrP. The green structure indicates canine Sho.

As 70_71DelAA is located in the interaction region of Sho with PrP, we assessed the impact of 70_71DelAA on the protein–protein interaction of canine Sho with canine PrP (Figure 5). A different binding conformation was observed in the complex of the protein–protein interaction of canine PrP with canine Sho carrying wild-type and 70_71DelAA alleles. Although the canine Sho carrying wild-type and 70_71DelAA alleles have similar 3D structures of protein (Figure 4), the TM-align (0.64840) and RMSD (3.74 Å) scores indicate that there are some structural differences between canine Sho carrying wild-type and 70_71DelAA alleles (Supplementary Table 3). As expected, the binding free energy of canine PrP with canine Sho carrying the wild-type allele (−39.96 kcal/mol) is lower than that of canine PrP with canine Sho carrying the 70_71DelAA allele (-22.53 kcal/mol). We also observed the difference in TM-align and RMSD score between canine Sho carrying wild-type allele (TM-align score: 0.23865; RMSD: 3.92 Å) and canine Sho carrying 70_71DelAA allele (TM-align score: 0.24947; RMSD: 3.74 Å) (Supplementary Table 4), indicating that binding complexes have structural differences according to the Indel polymorphism. In addition, we investigated the conformational difference of the PrP-Sho complex in other species (Supplementary Figure 1) and observed that the PrP-Sho complex of each species has similar conformation but a different binding free energy.

## Discussion

In the present study, we found only one insertion/deletion polymorphism in the ORF of the canine *SPRN* gene. Previous studies have reported that prion-susceptible animals, including cattle, sheep, cats and goats, have highly polymorphic *SPRN* genes (11, 12, 21–26). In contrast, a prion-resistant animal, the horse, has one polymorphism of the *SPRN* gene (27, 28). The number of prion-resistant animals is adequate to find the rare genetic polymorphisms with <1% frequency, even though genetic polymorphisms have been explored only in a relatively small sample size in prion-resistant animals compared to prion-susceptible animals. As genetic polymorphisms may affect the function and structure of Sho, the difference in the number of these polymorphisms is noticeable. Future research is needed on the relationship between the number of *SPRN* polymorphisms and the susceptibility since pathogenic mutations are more likely to occur in highly polymorphic *SPRN* genes.

We also found three insertion/deletion polymorphisms located downstream of the *SPRN* gene. However, because the structure of the *SPRN* gene has not been confirmed, further annotation of these 3 *SPRN* polymorphisms is not available. Thus, it is highly necessary to further analyze the downstream region of the *SPRN* gene using 3′ rapid amplification of cDNA ends (RACE) to investigate the impact of the 3 *SPRN* polymorphisms. We observed strong LD between c.444+30_444+31InsGGCCCCC and c.444+31_444+32InsGGCCCC (Table 2). This indicates that LD block is present between these 2 *SPRN* polymorphisms.

As Sho was predicted to have an unstructured 3D structure, the whole 3D structure of Sho has not been determined thus far. Thus, we analyzed the whole 3D structures of Sho in prion-related species. Notably, all species have a similar 3D structure with 2 α-helixes linked with the coil (Figure 4). This indicates that a significant difference in the 3D structure of Sho was not observed between prion-susceptible and prion-resistant animals.

In the present study, the canine *SPRN* polymorphism found in the ORF is located in the interaction region of Sho with PrP. Thus, we investigated the difference in protein–protein interactions according to alleles of the canine *SPRN* polymorphisms. Notably, we found different binding complexes of canine PrP with canine Sho according to *SPRN* polymorphisms (Figure 5). In addition, the binding free energy of Sho and PrP is also different according to *SPRN* polymorphisms. The canine Sho with 70_71DelAA allele showed higher binding free energy compared to wild-type canine Sho (Figure 5). This result indicates that the canine Sho with 70_71DelAA allele has an unstable complex compared to wild-type Sho. In addition, a complex generated by canine PrP and Sho with the 70_71DelAA allele differs compared to the interaction of that with wild-type canine Sho, and to those shown in other species (Figure 5, Supplementary Figure 1). All interactions seem to proceed between the α-helical motifs from PrP and Sho, except the interaction of canine Sho with 70_71DelAA allele and canine PrP, where PrP appears as interacting with the unstructured region of Sho. However, the role of this polymorphism in pathology of prion diseases is elusive. Further bioassay using prion-infected dogs carrying the wild-type and 70_71DelAA alleles of canine *SPRN* gene is needed to confirm the role of the this polymorphism.

In addition to prion diseases, Sho has various physiological roles, including the development and differentiation of embryos and mammary glands. The *SPRN*-knockout pups showed augmented embryonic lethality rates. Furthermore, *SPRN* knockout female mice showed a lactation defect (29–31). As the difference in Sho according to *SPRN* polymorphisms was identified, further investigation of the impact of the polymorphisms on the physiology of the embryo and mammary gland is highly desirable in the future.

Although we predicted the 3D structure of Sho using AlphaFold, the coil between the two α-helices has a low confidence (pLDDT < 70) in all species. In addition, conserved putative interaction sites with PrP (codons 63–77 in dogs) also showed low confidence (pLDDT < 50). To confirm the 3D structure of Sho and obtain reliable results for comparison of putative interaction site with PrP, the identification of Sho structure using nuclear magnetic resonance (NMR) and X-ray crystallography is needed in the future. Furthermore, while we investigated the 3D structure of Sho in several species, we did not analyze the impact of prion-resistant polymorphisms found in prion-susceptible species. Future research on the impact of prion-resistant genetic polymorphisms on the 3D structures of the Sho and PrP-Sho binding complex is highly desired.

## Conclusion

In summary, we identified four insertion/deletion polymorphisms of the *SPRN* gene in 201 dogs and found a significant difference in the number of *SPRN* polymorphisms located on the ORF between prion-susceptible and prion-resistant animals. In addition, we found that the 3D structure of Sho is similar to that of prion-related animals. Furthermore, we found a significant difference in the protein–protein interaction of canine PrP with canine Sho according to *SPRN* polymorphisms. To the best of our knowledge, this is the first report of canine *SPRN* polymorphisms.

## Data availability statement

All other data supporting the findings of this study are available from the corresponding author on reasonable request.

## Ethics statement

The animal study was reviewed and approved by Institutional Animal Care and Use Committee (IACUC) of Jeonbuk National University.

## Author contributions

Y-CK and B-HJ conceived and designed the experiment, analyzed the data, and wrote the paper. Y-CK, H-HK, and A-DK performed the experiments. All authors read and approved the final manuscript. All authors contributed to the article and approved the submitted version.

## Funding

This work was supported by the National Research Foundation of Korea (NRF) grant funded by the Korea government (MSIT) (2021R1A2C1013213 and 2022R1C1C2004792). This research was supported by the Basic Science Research Program through the National Research Foundation (NRF) of Korea funded by the Ministry of Education (2017R1A6A1A03015876 and 2021R1A6A3A010864). Y-CK and H-HK were supported by the BK21 plus program in the Department of Bioactive Material Sciences.

## Conflict of interest

The authors declare that the research was conducted in the absence of any commercial or financial relationships that could be construed as a potential conflict of interest.

## Publisher's note

All claims expressed in this article are solely those of the authors and do not necessarily represent those of their affiliated organizations, or those of the publisher, the editors and the reviewers. Any product that may be evaluated in this article, or claim that may be made by its manufacturer, is not guaranteed or endorsed by the publisher.

## Supplementary material

Supplementary Figure 1 The tertiary structure and protein–protein interaction of PrP with Sho in humans, cattle, sheep, goats, red deer, and horses. **(A)** Human PrP (PDB ID: 5yj5) interaction with human Sho. **(B)** Bovine PrP (PDB ID: 1dx1) interaction with bovine Sho. **(C)** Ovine PrP (PDB ID: 1uw3) interaction with ovine Sho. **(D)** Caprine PrP (PDB ID: 3o79) interaction with caprine Sho. **(E)** Cervid PrP (PDB ID: 1xyw) interaction with cervid Sho. **(F)** Equine PrP (PDB ID: 2ku4) interaction with equine Sho. The purple structures indicate PrPs. The blue structures indicate Sho proteins.

Supplementary Table 1 Detailed information on dog breeds investigated in the present study. ^*^Mixed dogs are cross-breed dogs originating from breeding between a Maltese and a Toy Poodle. The origin of mixed dogs was confirmed by a pedigree document.

Supplementary Table 2 Detailed information on the Sho protein investigated in the present study.

Supplementary Table 3 Structural similarity of the wild-type Sho of dogs with Sho of several species using TM-align. RMSD, root mean square deviation.

Supplementary Table 4 Structural similarity of the canine PrP with canine Sho according to Indel polymorphism using TM-align. RMSD, root mean square deviation.
