# Supplementary figures and images for: Novel insertion/deletion polymorphisms and genetic features of the shadow of prion protein gene (SPRN) in dogs, a prion-resistant animal
Source: Front Vet Sci. 2022 Aug 2;9:942289. doi: 10.3389/fvets.2022.942289 (PMC9378991; doi:10.3389/fvets.2022.942289)

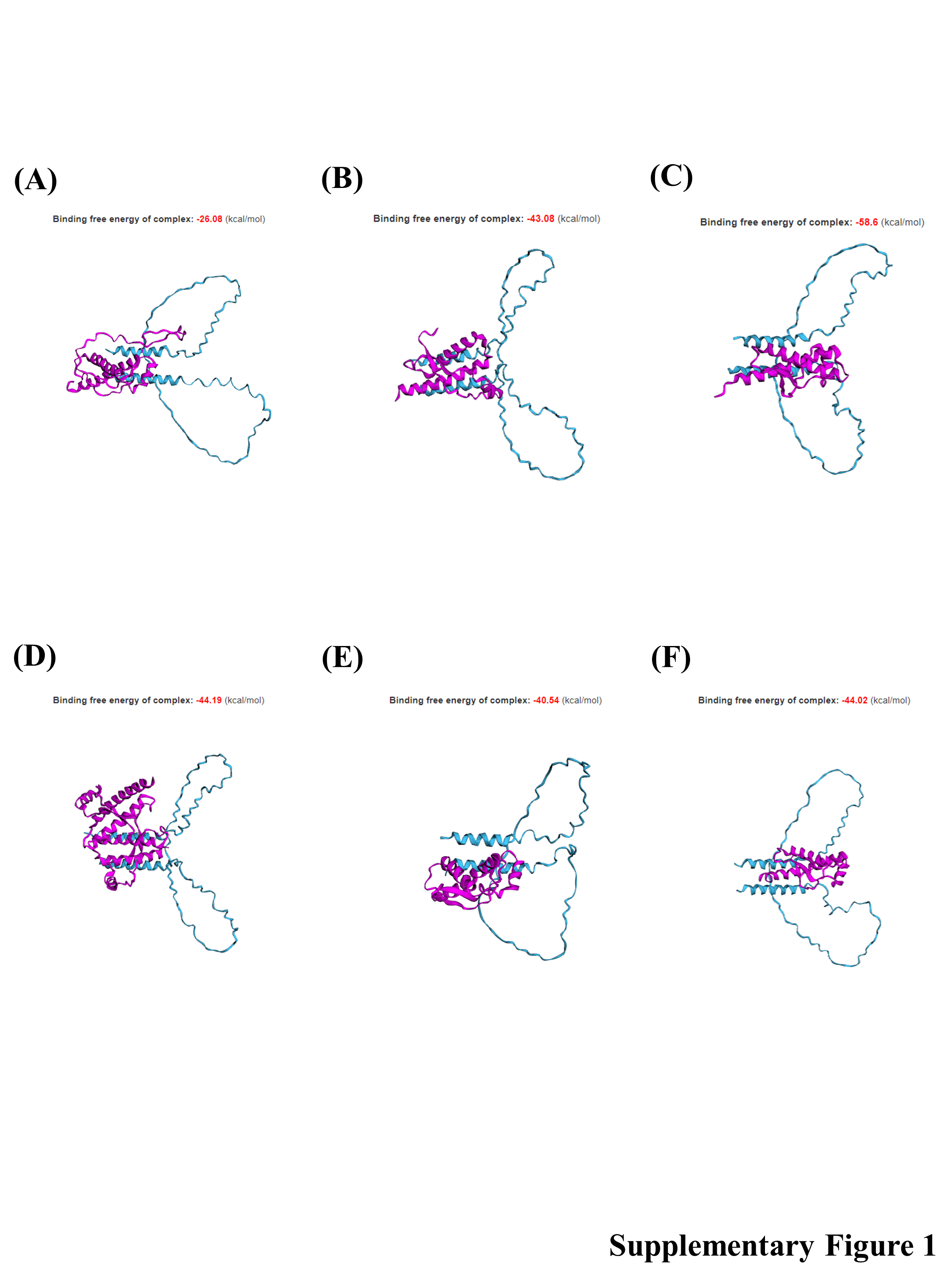

Supplement: Supplementary Figure 1 — The tertiary structure and protein–protein interaction of PrP with Sho in humans, cattle, sheep, goats, red deer, and horses. (A) Human PrP (PDB ID: 5yj5) interaction with human Sho. (B) Bovine PrP (PDB ID: 1dx1) interaction with bovine Sho. (C) Ovine PrP (PDB ID: 1uw3) interaction with ovine Sho. (D) Caprine PrP (PDB ID: 3o79) interaction with caprine Sho. (E) Cervid PrP (PDB ID: 1xyw) interaction with cervid Sho. (F) Equine PrP (PDB ID: 2ku4) interaction with equine Sho. The purple structures indicate PrPs. The blue structures indicate Sho proteins. [file Image_1.TIF]
