# Supplementary material for: Novel insertion/deletion polymorphisms and genetic features of the shadow of prion protein gene (SPRN) in dogs, a prion-resistant animal
Source: Front Vet Sci. 2022 Aug 2;9:942289. doi: 10.3389/fvets.2022.942289 (PMC9378991; doi:10.3389/fvets.2022.942289)
Supplement: Supplementary Table 1 — Detailed information on dog breeds investigated in the present study. *Mixed dogs are cross-breed dogs originating from breeding between a Maltese and a Toy Poodle. The origin of mixed dogs was confirmed by a pedigree document. [file Data_Sheet_1.docx]

| Breeds | Number, n |
| --- | --- |
| Maltese | 74 |
| Shih Tzu | 29 |
| Toy Poodle | 25 |
| Yorkshire Terrier | 19 |
| Pomeranian | 15 |
| Chihuahua | 11 |
| Schnauzer | 7 |
| Bichon Frise | 5 |
| Mixed dog* | 16 |
| Total | 201 |

**Supplementary Table 1** Detailed information on dog breeds investigated in the present study.

*Mixed dogs are cross-breed dogs originating from breeding between a Maltese and a Toy Poodle. The origin of mixed dogs was confirmed by a pedigree document.

**Supplementary Table 2** Detailed information on the Sho protein investigated in the present study.

| Common name | Scientific Name | Protein ID | Length | Identity |
| --- | --- | --- | --- | --- |
| Human | *Homo sapiens* | NP_001012526.2 | 151 | 122/151 (80.8%) |
| Cattle | *Bos taurus* | AAY83885.1 | 149 | 115/149 (77.2%) |
| Sheep | *Ovis aries* | NP_001156033.1 | 150 | 113/150 (75.3%) |
| Goats | *Capra hircus* | AGU17009.1 | 152 | 113/152 (74.3%) |
| Red deer | *Cervus elaphus* | ACF24724.1 | 149 | 112/149 (75.2%) |
| Horse | *Canis lupus familiaris* | XP_038296952.1 | 147 | 118/147 (80.3%) |
| Dog | *Equus caballus* | XP_023492126.1 | 147 | - |

**Supplementary Table 3** Structural similarity of the wild-type Sho of dogs with Sho of several species using TM-align.

| Species | TM-score | RMSD |
| --- | --- | --- |
| Human | 0.45032 | 4.59 Å |
| Cattle | 0.5253 | 4.50 Å |
| Sheep | 0.51838 | 4.32 Å |
| Goats | 0.48278 | 4.88 Å |
| Red deer | 0.34126 | 5.37 Å |
| Horse | 0.56489 | 4.45 Å |
| Dog (Indel allele) | 0.64840 | 3.74 Å |

RMSD: root mean square deviation

**Supplementary Table 4** Structural similarity of the canine PrP with canine Sho according to Indel polymorphism using TM-align.

| Protein | TM-score | RMSD |
| --- | --- | --- |
| Canine Sho (Wild type allele) | 0.23865 | 3.92 Å |
| Canine Sho(70_71DelAA allele) | 0.24947 | 3.74 Å |

RMSD: root mean square deviation
